# The Impact of Air Pollution on Residents’ Happiness: A Study on the Moderating Effect Based on Pollution Sensitivity

**DOI:** 10.3390/ijerph19127536

**Published:** 2022-06-20

**Authors:** Xuan Tian, Cheng Zhang, Bing Xu

**Affiliations:** 1 School of Economics, Nanjing University of Finance and Economics, Nanjing 210003, China; 1120200662@stu.nufe.edu.cn; 2 School of Finance, Nanjing University of Finance and Economics, Nanjing 210003, China; zhangcheng@nufe.edu.cn; 3 Wenzhou Base, National Institution for Finance & Development, Wenzhou Business College, Wenzhou 325015, China

**Keywords:** objective air pollution, subjective air pollution, happiness, pollution sensitivity

## Abstract

Promoting people’s happiness is a vital goal of public policy, and air pollution, as the focus of public opinion, is an important influencing factor of residents’ happiness. Although previous literature has explored the relationship between air pollution and happiness, the impact of pollution sensitivity on the relationship has so far received little attention. This paper uses the 2016 China Labor-force Dynamics Survey database (CLDS) to study the impact of air pollution on personal happiness and dissects the moderating effect of air pollution sensitivity from the stock and incremental perspectives. The results found that (1) there is an inverted U-shaped relationship between air pollution and residents’ happiness, such that happiness increases and then decreases with increasing air pollution. The PM10 concentration at the turning point is 119.69 μg/m^3^, which exceeds the national secondary standard limit (70 μg/m^3^) by 70.99% and is at the intermediate stage of mild pollution, exceeding the WHO recommended standard (20 μg/m^3^) by 498.45%, far higher than the international standard recommended level; (2) both air pollution stock sensitivity and incremental sensitivity have a significant positive moderating effect on the relationship between air pollution and happiness, and pollution sensitivity exacerbates the negative effect of air pollution on residents’ happiness by shifting the curve turning point to the left and steepening the curve shape; (3) in addition, the effect of air pollution on different groups is significantly heterogeneous, with lower-age and male groups more likely to have lower happiness due to air pollution; the positive moderating effect of pollution sensitivity is more significant in lower-age, female, and higher-income groups. Therefore, in order to enhance residents’ happiness, the government should not only improve air quality, but also focus on helping residents establish an appropriate subjective perception of air quality.

## 1. Introduction

With the rapid development of China’s economy, the overall welfare level of residents began to receive more and more attention. The latest World Happiness Report 2022 released by the United Nations shows that mainland China ranks 72nd out of 146 countries and regions participating in the survey, with the overall happiness of its residents at a medium level. As the world’s second-largest economy, China’s residents’ happiness index is lower than that of many developing countries, although its economic growth rate remains high year-round [1]. Happiness, as an important indicator reflecting people’s livelihood, has received extensive attention in psychology, sociology, economics and other disciplines. Early studies on happiness focused on individual characteristics, but individuals are in the structure of the collective and society, and it is not ideal to ignore social factors when studying happiness. In response, attention should be paid to well-being from a social perspective, to the influence of society on individuals, and to the public sphere of well-being [2].

Good air quality is the fairest public product and the most universal welfare of people’s livelihood. However, since the reform and opening up, China has created a miracle of economic growth, while the rough development model has brought serious air pollution problems. For this, in recent years, China has promulgated a series of air pollution control policies to gradually strengthen the strength, breadth and depth of air pollution control [3]. At present, the national air quality has improved in general, but some areas of air pollution have rebounded seriously, and the national air pollution problem is still not ideal. According to the “2020 Global Environmental Performance Index Report” jointly released by Yale University and other institutions, China’s environmental performance evaluation score is 37.3 points, ranking 120 out of 180 participating countries and regions in the world. The pollution situation is still difficult, and pollution control has a long way to go.

Existing literature has extensively explored the relationship between air pollution and happiness. Most of the existing literature verifies the negative impact of air pollution on happiness from a linear perspective [4,5,6,7,8]. Welsch [9,10,11,12], Luechinger [13] and Schmitt [14], based on annual average air pollution data at the national level, verified that air pollution significantly reduces the happiness of the population in each country. To improve the accuracy and matching of the data, Smyth et al. [15], Ferreira and Moro [4], and Levinson [5] also demonstrated the negative effect of air pollution on happiness using city-level pollution data. Zhang et al. [16], Li et al. [17], and Zheng et al. [18] precisely matched immediate air pollution with individual emotional states and found that air pollution reduced residents’ immediate happiness and increased the incidence of depressed mood. However, some literature suggests that there is no significant relationship between the two [19], or even a positive relationship [20,21,22].

**Figure 1:**
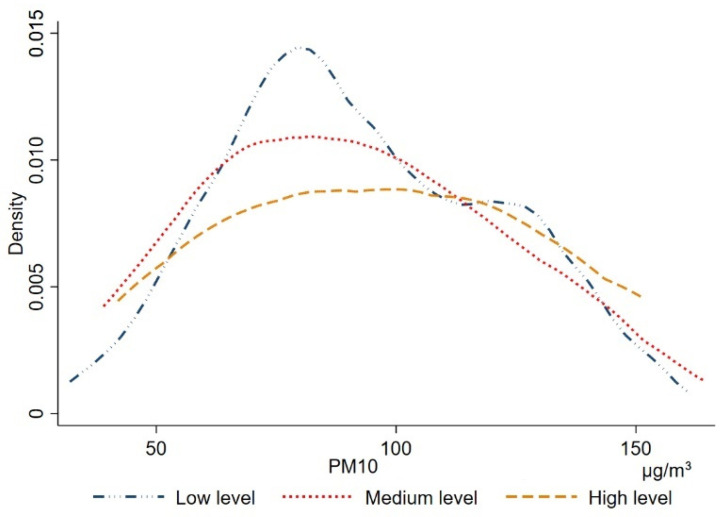
Kernel density map of objective air pollution distribution under different subjective air pollution levels.

Happiness is not only affected by objective air pollution, but people’s subjective evaluation of air pollution also affects happiness [23,24,25]. Rehdanz and Maddison [26], MacKerron and Mourato [27], and Welsch [11] used data from European and American countries and found that subjective air pollution has a significant negative effect on the residents’ happiness. Some studies have even shown that subjective air pollution has a greater degree of impact on residents’ happiness compared to objective air pollution [16,19]. However, there is a discrepancy between objective air pollution and subjective air pollution due to biases in people’s perceptions. Figure 1 plots the kernel density of objective air pollution distribution using subjective air pollution data from the 2016 China Labor Force Dynamics Survey Database (CLDS) and PM10 concentration data from the China City Statistical Yearbook. As shown in the figure, under each objective air pollution level, different levels of subjective air pollution levels are corresponding. When the PM10 concentration is less than 50 µg/m^3^, about 5% of the population believes that the air pollution is at a high level; at PM10 concentrations greater than 150 µg/m^3^, 0.6% of the residents still believe that the air pollution is at a low level, which further indicates the variability between subjective and objective air pollution.

Psychological studies show that the difference between subjective evaluation and objective levels is influenced by individual sensitivity, and the higher the sensitivity to something, the stronger the perception. Zheng et al. [20] and Matthew et al. [28] pointed out that the more sensitive a group is to air pollution, the higher the demand for air quality and the more likely they are to become dissatisfied by air pollution. It is inferred that air pollution sensitivity moderates the effect of air pollution on individual happiness. Much of the existing literature focuses on differences in pollution sensitivity across individuals [27,29,30], but little attention has been paid to how pollution sensitivity affects the relationship between air pollution and happiness. Whether over-sensitivity to air pollution leads to anxiety or lack of sensitivity to air pollution leads to ignorance of environmental issues, it is not conducive to the establishment of good environmental perceptions among residents. Therefore, it is crucial to explore the effects of pollution sensitivity on residents and help them establish correct environmental perceptions.

In view of this, this paper uses the 2016 CLDS database to explore the role played by air pollution sensitivity in the relationship between objective air pollution and happiness and further analyzes the heterogeneity of objective air pollution on happiness. The main contributions of this paper are as follows. Firstly, compared with previous scholars who have single-handedly explored how air pollution negatively affects happiness, this paper dissects the positive and negative effects of air pollution on happiness and uses Chinese data to verify the inverted U-shaped relationship between air pollution and happiness. Secondly, this paper constructs pollution sensitivity indicators in two dimensions: stock and increment. On the one hand, different objective pollution stock levels bring different subjective perceptions of air pollution. The difference between subjective air pollution and present objective pollution reflects people’s sensitivity to the stock of air pollution. On the other hand, changes in air quality over a period of time also affect residents’ subjective perception of air pollution. The difference between subjective air pollution and air pollution increment responds to the individual’s air pollution increment sensitivity. Third, this paper analyzes the moderating effect of pollution sensitivity on the relationship between air pollution and residents’ happiness, which enriches the research on the quadratic moderating effect in the field of happiness.

The rest of this paper is organized as follows. Section 2 analyzes the relevant theories and proposes hypotheses to be tested, Section 3 presents data sources, variable construction, and model construction, Section 4 analyzes the empirical results, Section 5 discusses the empirical results, and Section 6 summarizes the main conclusions and implications.

## 2. Theoretical Background and Hypotheses

**Figure 2:**
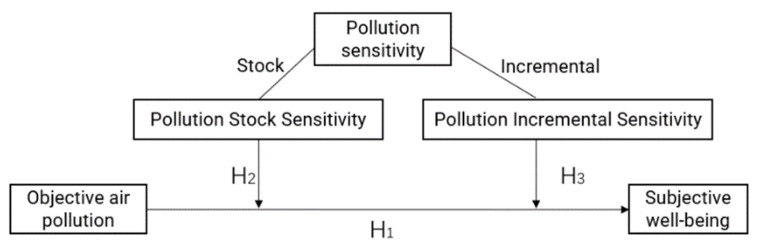
Theoretical model.

Two parts are discussed here. One is the relationship between air pollution and residents’ happiness, and the other is the impact of air pollution sensitivity on the relationship between air pollution and happiness. The theoretical model is shown in Figure 2.

### 2.1. The Effect of Objective Air Pollution on Residents’ Happiness

In the early stage of air pollution, there may be a positive relationship between air pollution and happiness. Firstly, according to the Environmental Kuznets Curve, economic growth and environmental pollution have an inverted U-shape relationship. In the early stages of economic development, the objective presence of air pollution is exchanged for economic growth, which leads to an increase in personal income at the micro level, thus compensating for the loss of air pollution to the residents, which in turn increases their comfort and psychological well-being [31,32,33,34]. Secondly, economic growth is accompanied by improvements in certain social factors at the macro level, including social capital, urban modernization, and infrastructure development, and these collateral effects can likewise enhance the residents’ happiness [35,36,37,38]. Finally, when the air pollution level is within the standard state range, it is less harmful to health and less likely to cause an impact on human senses such as vision and smell. At this time, people’s perception of air pollution is weaker and less concerning, and air pollution is less likely to have an impact on the human body. In summary, in the early stage of air pollution, air quality decline can instead enhance the sense of happiness.

However, according to the “Easterlin paradox”, the marginal effect of economic growth on happiness decreases gradually, and when the level of economic development exceeds a certain threshold, its effect on happiness stagnates [35,39,40]. At this point, the negative effects of air pollution on happiness come to the fore. On the one hand, air pollution can directly affect human emotions by affecting the earth’s atmosphere. Li et al. [17] used a psychophysical approach to test the relationship between the two, and the experimental results showed that air pollution significantly increases people’s negative emotions such as stress and depression and has a direct negative impact. On the other hand, air pollution also indirectly affects people’s happiness feelings through microscopic mechanisms. Objective air pollution increases the incidence of cardiovascular and respiratory diseases [41,42,43,44,45,46,47,48], which affects happiness by affecting residents’ health. Air pollution also reduces residents’ happiness by eroding transportation facilities and causing inconvenience to their lives [4,49].

To sum up, considering the contrast between the positive and negative effects of air pollution on residents’ happiness, this paper puts forward Hypothesis 1 (H1):

**Hypothesis** **1** **(H1).** *There is an inverted U-shaped relationship between residents’ happiness and air pollution. Residents’ happiness will increase first and then decrease with increasing air pollution.*

### 2.2. Moderating Effect of Air Pollution Stock Sensitivity on Air Pollution and Happiness

People’s sensitivity to air pollution stock varies, resulting in differences between objective air pollution levels and people’s subjective perceptions of air pollution levels. Klerck and Sweeney [50], Li et al. [23], and Gu et al. [51] pointed out that with the same objective air pollution stock, the more environmental protection knowledge residents have, the more they have a stronger perception of air pollution. Ye and Zhang [22] pointed out that there are differences in air pollution sensitivity among different income groups. Compared to low-income groups, middle- and high-income groups are more sensitive to air pollution and have higher subjective evaluations of air pollution indices. Different individuals not only differ in their pollution sensitivity due to objective characteristics such as age, gender, education, health status, income level, and area of residence [7,52,53], but also due to emotional factors, personality traits, and other subjective differences in pollution sensitivity due to differences in psychological traits such as emotional factors and personality traits [54].

Residents are more likely to have negative emotions psychologically when they are sensitive to existing air pollution, such as concerns about their health status, fears about their future living environment, and worries about their future quality of life. Zheng et al. [18] and Matthew et al. [28] constructed a sentiment-pollution elasticity index to respond to residents’ sensitivity to air pollution and found that residents living in different areas differed in their sensitivity to air pollution. Urban residents with air pollution levels at the lighter and heavier poles were more sensitive to air pollution, and air pollution-sensitive groups had a greater need for clean air and were more likely to have reduced happiness due to air pollution. Health status also affects individuals’ judgments of objective air pollution levels, with people with poorer health and older people being less resistant to air pollution, more sensitive to air pollution, and more likely to be anxious due to air pollution [29,55,56,57]. Thus, air pollution stock sensitivity may enhance the negative effect of air pollution on happiness:

**Hypothesis** **2** **(H2).** *Stock sensitivity to air pollution has a moderating effect on the inverted U-shaped curve relationship between air pollution and happiness. The stronger the stock sensitivity to air pollution, the greater the negative effect of air pollution on residents’ happiness.*

### 2.3. Moderating Effect of Air Pollution Incremental Sensitivity on Air Pollution and Happiness

Research on subjective perceptions has mostly been approached from a stock perspective, but increments also have a crucial impact on subjective feelings. For example, in the exploration of the relationship between income and happiness, it was found that stock income has a limited effect on happiness, but incremental income can significantly enhance happiness [58]. In contemporary society, middle-aged and elderly people at the same income level usually have higher satisfaction with their living conditions. Having experienced China’s take-off after the reform and opening-up, the elders have made the leap from poverty to affluence, felt the progress of the social environment, and are greatly satisfied with their living conditions today. However, young people have lived an affluent life since childhood and are less satisfied with the affluent life. Similarly, people’s subjective judgment of air pollution is affected not only by the stock of air pollution, but also by the increment of air pollution. Under the same conditions, the higher the level of incremental air pollution, the stronger the residents’ subjective judgments of air pollution. The effect of incremental pollution sensitivity on happiness has not been studied yet. According to the moderating effect mechanism of stock pollution sensitivity on well-being, this paper speculates that incremental pollution sensitivity will also strengthen the negative effect of air pollution on well-being. In view of this, this paper proposes the following assumptions:

**Hypothesis** **3** **(H3).** *Incremental sensitivity to air pollution has a moderating effect on the inverted U-curve relationship between air pollution and happiness. The stronger the incremental sensitivity to air pollution, the greater the negative effect of air pollution on residents’ happiness.*

## 3. Materials and Methods

### 3.1. Data Sources and Processing

The micro-data used in this paper come from the 2016 CLDS database, which contains tracking and cross-sectional data at 3 levels of Chinese society: the labor force, household and community. The 2016 CLDS data sample covers 29 provinces, 158 cities, 11,631 households and 21,086 individual labor forces in China, which is nationally representative. This paper also matches the macro socio-economic and environmental pollution indicators of 113 cities in 2015 with the individual micro-data in 2016 CLDS, and the city data comes from the China Urban Statistical Yearbook, China Environmental Statistical Yearbook and the National Bureau of Statistics. This paper cleans the data by removing omissions and missing values as well as data matching, and the final valid sample size of individual labor is 7143.

### 3.2. Variable Construction

The explained variable in this paper is residents’ happiness, which comes from question I7.6.1 of the 2016 CLDS individual survey questionnaire, which asks, “In general, do you think you are happy with your life?”. Respondents were asked to choose from “very unhappy”, “unhappy”, “average”, “happy” and “very happy”, whose value is 1 to 5, respectively. The higher the value, the higher the happiness.

The core explanatory variable of this paper is objective air pollution, and the adoption of urban PM10 concentration as an indicator of air pollution is mainly based on the following reasons: Firstly, the frequent occurrence of hazy weather has increased people’s concern about particulate pollutants, which are the primary pollutants affecting air quality in China. Secondly, PM10 has the greatest impact on atmospheric visibility, which brings inconvenience to people’s travel and greatly increases traffic accidents. Thirdly, PM10 is likely to cause harm to human health, and PM10 significantly increases the incidence of human respiratory diseases, chronic obstructive pulmonary diseases, and cardiovascular diseases [59,60].

The control variables were selected as individual characteristic variables and city characteristic variables. Among the individual characteristic variables, this paper mainly controlled for the respondents’ gender, age and age-squared, marital status, political outlook, whether they paid pension insurance, whether they paid medical insurance, household registration location, personal income, trust in the surrounding environment, subjective social status, education level, and self-rated health status; the city characteristic variables were selected as GDP per capita, population density, and public finance expenditure ratio.

The moderating variables are stock pollution sensitivity and incremental pollution sensitivity. In this paper, the difference between subjective air pollution and objective air pollution is used as an indicator to measure stock pollution sensitivity. In order to eliminate the dimensional relationship between the variables and make the data comparable, this paper standardizes the deviation between subjective air pollution and objective air pollution so that the data are linearly transformed, and the results are mapped to the interval of [0, 1]. Then, the constructive equation of spatial air pollution sensitivity *M*1 is expressed in the following Equation (1). When *M*1 is positive, the standardized subjective air pollution is greater than the objective air pollution, and the residents overestimate the stock air pollution level and have a higher stock air pollution sensitivity.

(1) $$M1=\frac{X_{\mathrm{subjective}}-MIN_{\mathrm{subjective}}}{MAX_{\mathrm{subjective}}-MIN_{\mathrm{subjective}}}—\frac{X_{\mathrm{objective}}-MIN_{\mathrm{objective}}}{MAX_{\mathrm{objective}}-MIN_{\mathrm{objective}}}$$

In this paper, the rate of change of air pollution for two years, 2013–2015, is taken to measure the incremental air pollution level. The difference between subjective air pollution and air pollution change rate responds to the incremental air pollution sensitivity. Again, the subjective air pollution and the rate of change of air pollution are normalized by the deviation, and the equation for calculating the incremental air pollution sensitivity *M*2 is expressed by Equation (2). The larger the value of *M*2, the higher the incremental pollution sensitivity of the residents.

(2) $$M2=\frac{X_{\mathrm{subjective}}-MIN_{\mathrm{subjective}}}{MAX_{\mathrm{subjective}}-MIN_{\mathrm{subjective}}}—\frac{X_{\mathrm{rate}\;\mathrm{of}\;\mathrm{change}}-MIN_{\mathrm{rate}\;\mathrm{of}\;\mathrm{change}}}{MAX_{\mathrm{rate}\;\mathrm{of}\;\mathrm{change}}-MIN_{\mathrm{rate}\;\mathrm{of}\;\mathrm{change}}}$$

Table 1 shows the descriptive statistics of each variable. The mean value of happiness in the sample is 3.86, which is generally at a high level. Among them, 26% are “very happy”, 42% are “happy”, 26% are “average”, 4% are unhappy, and 2% are very unhappy. Concentrations range from 39–164 µg/m^3^, with large differences in air quality between cities. The mean values of stock pollution sensitivity and incremental pollution sensitivity are less than 0, indicating that residents are generally less sensitive to air pollution and slightly more sensitive to incremental pollution than stock pollution.

### 3.3. Model Construction

In this paper, the dependent variable “happiness” is an ordered discrete variable, and the Ordered Probit model is used to estimate the effect of air pollution on happiness with reference to Levinson’s [5] study. The benchmark regression model in this paper is as follows:

(3) $$Happniss_{i}=\beta_{0}+\beta_{1}air_{j}+\beta_{2}X_{i}+\beta_{3}Y_{j}+\varepsilon_{i}$$

(4) $$Happniss_{i}=\beta_{0}+\beta_{1}air_{j}+\beta_{2}air_{j}{}^{2}+\beta_{3}X_{i}+\beta_{4}Y_{j}+\varepsilon_{i}$$

where the explained variable $happiness_{i}\;$indicates the happiness of respondent i; $air_{j}\;$indicates the degree of air pollution in prefecture-level city *j*, which is represented by PM10 concentration in this paper; $X_{i}$ is the set of individual characteristic variables, and$\;Y_{j}$ is the set of city characteristic variables. Equation (3) contains only the primary term of air pollution, and Equation (4) contains the primary and secondary terms of air pollution to examine the nonlinear relationship between air pollution and happiness.

Equation (4) presents an inverted U-shaped relationship with symmetry on both sides of the turning point. Considering that the actual effect of PM10 on happiness is not symmetrical before and after the turning point, this paper builds a dummy variable model of the critical index on the basis of Equation (4) to examine the asymmetric change trend on both sides of the turning point. By setting $air_{j}{}^{*}$ as the turning point of the inverted U-shaped curve of air pollution concentration, the dummy variable of Equation (5) and the regression model of Equation (6) are constructed. Then, the coefficients $\beta_{1}$ reflect the degree of impact of air pollution on happiness before reaching the threshold, and the coefficients $\beta_{1}+\beta_{2}\;$reflect the degree of impact of air pollution on happiness after reaching the threshold.

(5) $$D_{air}=\{\begin{array}{c} 0,\;air<air_{j}{}^{*}\\ 1,\;air\geq air_{j}{}^{*} \end{array}$$

(6) $$Happniss_{i}=\beta_{0}+\beta_{1}air_{j}+\beta_{2}\left(air_{j}-air_{j}{}^{*}\right)\times D_{air}+\beta_{3}X_{i}+\beta_{4}Y_{j}+\varepsilon_{i}$$

Meanwhile, in order to examine whether the stock and incremental pollution sensitivities are moderating variables affecting the relationship between air pollution and happiness, on the basis of Equation (4), the interaction terms of air pollution, stock pollution sensitivities, and incremental pollution sensitivities after centralization are added to the equation to construct the moderating effect model:

(7) $$\begin{array}{c} Happniss_{i}=\beta_{0}+\beta_{1}air_{j}+\beta_{2}air_{j}{}^{2}+\beta_{3\;}air_{j}\times M+\beta_{4}air_{j}{}^{2}\times M+\beta_{5}M+\beta_{6}X_{i}\\ \;+\beta_{7}Y_{j}+\varepsilon_{i}\; \end{array}$$

where *M* is the moderating variable, which indicates the air pollution sensitivity, and $\varepsilon_{i}$ is a random error term. Referring to Haans et al. [61] for the test of the moderating effect of the U-shaped relationship, a hierarchical regression is conducted. If the coefficients $\beta_{1},\;\beta_{2},\;\beta_{3},\;\beta_{4}$ are all significant, the moderating effect is established.

## 4. Results

### 4.1. Baseline Regression

Model 1 in Table 2 reports the results of the linear regression of air pollution and happiness, and the PM10 coefficient is significant at the 5% level, indicating a significant positive relationship between air pollution and happiness (b = 0.001, se = 0.001). Model 2 is the nonlinear regression result after adding the quadratic term of air pollution. This paper draws on the three-step method proposed by Lind and Mehlum [62] to test the hypothesis of an inverted U-shaped relationship between air pollution and happiness: the first step requires that the coefficient $\beta_{1}$ of the primary term of the explanatory variable is significantly positive and the coefficient $\beta_{2}$ of the secondary term is significantly negative. In the regression results of model 1, the coefficient of PM10 is positive, which is significant at the 5% level (b = 0.008, se = 0.003). The coefficient of PM10^2^ is negative, which is significant at the 5% level (b = 3.342 × 10^−5^, se = 1.661 × 10^−5^), which satisfies condition one. The second step requires the slope of the curve to be negative when the explanatory variable takes the minimum value and positive when it takes the maximum value. Substituting the minimum value of 39 and the maximum value of 164, $Happniss_{i_{PM10_{min}}}^{\prime }$ is 0.006 and $Happniss_{i_{PM10_{max}}}^{\prime }$ is −0.002, which satisfies condition two. The third step requires that the turning point takes a value that lies within the range of values of the explanatory variables. Since the turning point of the curve is the value taken when the slope of the curve $Happniss_{i}^{\prime }$ = 0, $air_{j}{}^{*}=-\beta_{1}/2\beta_{2}=$ 119.69 µg/m^3^, which is within the range of values, satisfies condition three. Therefore, air pollution and residents’ happiness show an inverted U-shaped relationship, and residents’ happiness rises and then falls with the increase in air pollution level, and hypothesis 1 is verified.

Among the control variables, the coefficient of age is negative (b = 0.066, se = 0.007), while the coefficient of the squared term of age is positive (b = 0.072, se = 0.008), indicating a positive U-shaped relationship between individual age variables and happiness, which is consistent with most literature findings [63,64]. As age increases, happiness tends to first decrease and then increase, with a low point of happiness around age 47. The low happiness index in midlife may be related to higher life stress and work intensity. In terms of gender, women’s happiness is significantly higher than men’s (b = 0.118, se = 0.027), probably due to the fact that Chinese men generally bear more work pressure and family expectations, or there may be significant psychological differences between genders. The coefficient of marital status is significantly positive (b = 0.311, se = 0.043), and marriage is often considered an essential part of a happy life. Marriage can achieve economies of scale through the complementarity of goods and other inputs. Married [65] individuals are generally healthier, wealthier, and have a lower risk of depression. The coefficient of education level is significantly positive (b = 0.068, se = 0.014), and education not only enhances happiness by changing individuals’ cognitive abilities, but also improves one’s economic income and social status by acquiring economically valuable knowledge and skills, which in turn enhances happiness. Income is positively correlated with happiness, which verifies the importance of economic base on happiness. Social trust is positively correlated with happiness (b = 0.169, se = 0.016). Individuals’ sense of trust in the external world is both the basis of individual security and an important safeguard against anxiety and generates happiness. Religious beliefs (b = 0.049, se = 0.039), GDP per capita (b = 0.037, se = 0.039), and public expenditure ratio (b = 376, se = 0.274) have no significant effects on happiness.

Model 3 shows the results of the phased regression before and after the turning point, and the coefficient of PM10 is positive before reaching the turning point (b = 0.003, se = 0.001). After reaching the turning point, the coefficient of PM10 is negative (b = −0.004, se = 0.002). Both are significant at the 1% level. The results are generally consistent with model 2, although there is some variability, which verifies the accuracy of the inverted U-shaped curve. Additionally, the magnitude of the coefficient shows that the negative effect of air pollution on happiness after reaching the turning point is greater than the positive effect on happiness before reaching the turning point.

### 4.2. Test of Moderating Effect

Table 3 presents the test results of this paper on Hypothesis 2 and Hypothesis 3. The interaction term coefficients of PM10, PM10^2^ and stock pollution sensitivity (b = 0.023, se = 0.007; b = −1.223 × 10^−4^, se = 3.642 × 10^−5^) and incremental pollution sensitivity (b = 0.037, se = 0.008; b = −1.952 × 10^−4^, se = 4.304 × 10^−5^) are all significant at the 1% statistical level. It indicates that pollution sensitivity has a significant moderating effect on the relationship between air pollution and happiness. Referring to the test method of Haans et al. [61] for the U-shaped relationship, this paper analyzes the moderating effect of pollution sensitivity in terms of both the steepening or flattening of the curve shape and the direction of movement of the turning point.

In the results of the moderating effect in Table 3, model 1 is the moderating effect of stock pollution sensitivity with a negative sign; that is, the moderating effect shifts the turning point of the inverted U-shaped curve to the left, and the greater the stock pollution sensitivity is, the lower the concentration of nodes where air pollution reduces happiness will be. β4 is significantly negative, and the moderating effect makes the shape of the inverted U-shaped curve steeper. Then, the stock pollution sensitivity leads to the negative effect of air pollution on happiness being stronger. Therefore, H2 is verified.

Model 2 is the moderating effect of incremental pollution sensitivity, and the results are similar to model 1. The empirical results also show that the incremental pollution sensitivity precipitates the turning point of the inverted U-shaped relationship between air pollution and happiness and strengthens the negative effect of air pollution on happiness. Therefore, H3 is verified.

Since the moderating effect of the inverted U-shaped curve shows a symmetric trend on both sides of the turning point, to verify that the change in curve shape after the turning point is not a mapping of the change before the turning point, model 3 and model 4 are tested separately for the effect of pollution sensitivity after the turning point. The results show that both PM10 and interaction term coefficients are significantly negative, and both stock and incremental pollution sensitivity play a positive moderating role in the effect of air pollution on happiness. The greater the residential air pollution sensitivity is, the stronger the negative effect of air pollution on happiness will be. Therefore, H2 and H3 are further verified.

### 4.3. Heterogeneity Analysis

Considering that the moderating effect of air pollution sensitivity on air pollution and residents’ happiness may differ among different groups, this paper analyzes the effect of air pollution on happiness and the moderating effect of pollution sensitivity by grouping according to different ages, genders and incomes, and the specific results are shown in Table 4, Table 5 and Table 6.

Table 4 presents the heterogeneity analysis for different age stages. The columns of model 1 and model 4 show that the relationship between air pollution and the happiness of residents in different age groups is inverted U-shaped, and the inverted U-shaped turning point of 115.21 μg/m^3^ in the low age group is earlier than that of 116.84 μg/m^3^ in the high age group, which means that the low age group will have an earlier decrease in happiness due to air pollution. The lower age group will have a lower sense of happiness due to air pollution earlier. Models 2, 3, 5 and 6 show the differences in the moderating effects of incremental and stock pollution sensitivity on air pollution and residents’ happiness. For the older group, the moderating effect of pollution sensitivity on both is not significant; this group mostly comes from the era of material collapse and pays more attention to material life enrichment while relatively ignoring the air pollution problem and being less sensitive to air pollution. For the younger group, both stock and incremental pollution sensitivity have a significant positive moderating effect. This group is growing up in a well-off environment, with diversified needs and a more urgent desire for a better life, and having high requirements for environmental protection and the environment they live in. Moreover, the young group will receive more information on the Internet and understand more clearly the hazards related to air pollution, so they are more sensitive to air pollution; thus, air pollution sensitivity will reduce the happiness of the young group.

Table 5 shows the heterogeneity analysis for males and females separately. The main effect was significant at the 1% level for males and at the 10% level for females. The curve turning point was 106.92 μg/m^3^ for males and 120.42 μg/m^3^ for females, and the pollution threshold for men to reduce their happiness due to air pollution was much earlier than that of women. In China, men are more engaged in productive labor, spend more time in the air, are more exposed to the hazards of air pollution, and are thus more likely to have reduced happiness due to air pollution. However, the moderating effect of pollution sensitivity in the male group was not significant, while there was a significant positive moderating effect of both stock and incremental pollution sensitivity in the female group. It has been shown that women have more sensitive perceptions than men [66,67] and are more likely to have greater mood swings. Women who are sensitive to the environment may have increased concerns about their health and fears of unsuitable living conditions. Therefore, it is more likely to affect happiness because of sensitivity to pollution.

Table 6 presents a heterogeneity analysis for different income groups. There is no significant difference between the main effects of the low-income group and the high-income group, and both of them have a significant inverted U-shaped relationship. The turning points of the curves are both around 112.00 μg/m^3^. The moderating effect of the low-income group is not significant, while the air pollution sensitivity of the high-income group has a significant positive moderating effect, and the pollution sensitivity increases the negative effect of air pollution on happiness. This indicates that the low-income group pays more attention to basic public goods such as the economy, education and health care and does not further reduce happiness due to sensitivity to air pollution. The high-income group, on the other hand, focuses more on higher-level needs other than the economy, knows the importance of environmental protection, has a higher level of awareness of environmental issues, and pays more attention to environmental quality. Therefore, high-income groups with sensitivity to air quality are prone to negative emotions due to air pollution.

### 4.4. Robustness Test

To improve the reliability and credibility of this study, this paper analyzes the robustness of the results from three aspects: a variable substitution test, a model substitution test, and a bilateral shrinkage tail test.

Firstly, a variable substitution test was conducted to examine the robustness of the regression results by replacing the explained variable and the explanatory variable separately. The variable of happiness was replaced from question I7.6.1, “Do you think you are happy in your life?” in the CLDS individual questionnaire to question I7.6.2, “Are you satisfied with your life situation?”. To examine the effect of different air pollutants on happiness, PM10 was replaced with SO_2_ and NO_2_. Model 1 and model 2 in Table 7 show the results of replacing the core variables. Similar to the PM10 regression results, the relationship between SO_2_ and NO_2_ and happiness is an inverted U-shaped curve, with the turning point of 65.32 µg/m^3^ for SO_2_ and 41.64 µg/m^3^ for NO_2_, and the turning points are within the range of values. Models 3–5 are the results of replacing the explained variables, and the estimated coefficients, which differ only slightly in magnitude and maintain the same sign, are all statistically significant, indicating that the results of hypothesis testing are universal.

In order to test whether the estimation results are sensitive to the estimation method, this paper performs a robustness test by replacing the regression method. It has been argued that ordinary least squares estimation and Ordered Probit estimation methods are not superior or inferior and OLS have more explanatory power. Probit models are similar to Logit models, and many scholars have also used Ordered Logit models to study happiness [68,69]. Thus, in this paper, the model regression results are tested using OLS regression and Ordered Logit regression. Table 8 models 1 and model 2 show the regression results of model substitution. The results of the three regressions are roughly the same, and they all present the results that happiness increases and then decreases with increasing air pollution.

Due to the large differences in pollution in different cities and the existence of extreme values of the variables, this paper conducts a bilateral shrinkage tail test on the 5% quantile for the core variable PM10 concentration, and model 3 in Table 8 shows the test results, with the turning point slightly shifted backward, but still within the range of PM10 concentration taken, and still in a significant inverted U-shaped curve.

## 5. Discussion

### 5.1. Air Pollution and Happiness

Before the turning point, economic growth in exchange for air quality improves residents’ happiness; after the turning point, air pollution seriously endangers physical and mental health, disturbs residents’ daily life, and causes happiness to decline. The PM10 concentration at the turning point is 119.69 µg/m^3^, which is in the 75th percentile of the total sample PM10 concentration. The annual average secondary standard limit value of PM10 specified in China’s ambient air quality standards (GB3095-2012) is 70 µg/m^3^, and the PM10 concentration at the turning point exceeds the limit value by 70.99%, which is at the intermediate stage of mild pollution. The WHO’s recommended standard for PM10 concentration is 20 μg/m^3^, and the PM10 concentration at the turning point exceeds the recommended standard by 498.45%, which is much higher than the baseline recommended level.

Although the turning point of air pollution in this paper far exceeds the world air pollution standards, it is reasonable under the special national conditions of China. First of all, the extremely high level of turning point reflects the emphasis on economic benefits and contempt for the harm of air pollution. China is in an era of rapid economic growth and wealth accumulation, and people pay more attention to economic benefits than to the harm of air pollution. Therefore, in most cases, the economic benefits of air pollution outweigh the various harms caused by air pollution, so the overall effect of air pollution on happiness is positive. We speculate that with the further development of China’s economic level, after people’s material wealth is greatly satisfied, the turning point of air pollution’s effect on happiness will be earlier. Second, China’s air pollution levels are at a high level all year round, and people have become accustomed to high levels of air pollution. Therefore, the negative impact of air pollution hazards on the well-being of the Chinese people is small, and the turning point is high. Finally, the Chinese government’s publicity on the harm of air pollution is insufficient, and the Chinese people’s awareness of the importance of air pollution is low, which leads people to underestimate the harm of air pollution and delays the turning point of air pollution on happiness.

A large number of international studies have discussed the relationship between air pollution and happiness based on data from Australia, Spain, Germany, the United Kingdom, India, Japan, South Korea, China and other countries. However, most of the existing studies assume that there is a monotonic relationship between air pollution and happiness [9,10,11,70,71,72,73,74,75], but air pollution at different stages may have different directions and different degrees of influence on happiness. Air pollution is often generated along with industrial development and urban modernization, and people both loathe the many hazards generated by air pollution and enjoy the economic benefits it brings. Therefore, the effect of air pollution on happiness can be decomposed into two effects, positive and negative. When the concentration of air pollution is low, people perceive less pollution and pay less attention to it, and economic growth brought about by declining air quality, and improved quality of life, urban modernization, and social capital growth due to economic growth [76] increase residents’ happiness; when the degree of air pollution continues to deepen to a certain critical value, the negative effects such as health hazards, stimulating human senses, reducing air visibility and hindering daily travel rise sharply. At this point, the marginal effect of clean air is greater than the marginal benefit of economic growth, and the happiness of the population decreases due to air pollution. Therefore, the impact of air pollution on happiness is subject to the contrast of positive and negative forces, and the relationship between the two is an inverted U-shaped relationship.

### 5.2. Modulation of Pollution Sensitivity

Consistent with the hypothesis, both incremental and stock pollution sensitivity positively moderate the relationship between air pollution and well-being (H2 & H3). Pollution sensitivity shifts the curve inflection point to the left and makes the curve shape steeper, thus exacerbating the negative effect of air pollution on well-being.

Air pollution sensitivity varies from person to person, resulting in differences in the degree of impact of the same air pollution level on the well-being of different individuals. Pollution sensitivity can cause deviations between residents’ subjective perceptions of pollution levels and actual pollution levels, leading to a mismatch between high pollution and low perceptions and low pollution and high perceptions. Residents’ pollution sensitivity is closely related to the level of environmental information disclosure by the government. The government should strengthen the effectiveness, timeliness, accuracy and symmetry of environmental information disclosure to ensure that residents can effectively understand the incremental and stock levels of pollution, reduce the bias of residents’ perception of pollution, and maintain a reasonable and moderate sensitivity to pollution.

## 6. Conclusions and Implications

### 6.1. Conclusions

There are great differences in the pollution sensitivity of different individuals, and pollution sensitivity will have a greater impact on the relationship between air pollution and happiness. However, so far, few studies have examined the effect of pollution sensitivity on the relationship between air pollution and well-being, especially when pollution sensitivity is used as a moderating variable. Using the 2016 CLDS database with urban air pollutant PM10 concentration data, this paper examines the impact of air pollution on personal happiness using the Ordered Probit model, Ordered Logit model, and OLS model and dissects the moderating effect of air pollution sensitivity from the stock and incremental perspectives. The study finds the following four conclusions.

Firstly, objective air pollution has an inverted U-shaped relationship with residents’ happiness, and this nonlinear relationship implies that the relationship depends on the effects of both economic growth and pollution hazards. That is, when the air pollution level is low, the economic growth brought by pollution dominates the growth of residents’ happiness, and their happiness gradually increases with the increase of air pollution. When the air pollution reaches 119.69 µg/m^3^, the harm of air pollution to residents dominates, leading to a decrease in happiness with an increase in air pollution. Secondly, the relationship between stock and incremental air pollution sensitivity has a significant moderating effect on the relationship between the two, and air pollution sensitivity shifts the turning point of the curve to the left and becomes steeper, i.e., air pollution sensitivity has a significant positive moderating effect on the relationship between air pollution and happiness. Again, there is heterogeneity in the effect of air pollution on happiness. The negative effect of air pollution on happiness was stronger in the lower-age male group. Finally, the positive moderating effect of air pollution sensitivity on the inverted U-shaped curve also differed across groups. The moderating effect was more significant among the lower age, female, and higher-income groups, and the stronger the pollution sensitivity is, the stronger the negative effect of air pollution on the happiness of such groups will be.

### 6.2. Practical Implications

This paper has important policy implications for setting air pollution control goals, assessing air pollution control performance, and guiding people to establish correct environmental concepts. In order to effectively improve air quality and enhance residents’ subjective sense of happiness, firstly, the government should combine ecological carrying capacity and economic carrying capacity when setting air pollution control targets [77] and take promoting people’s happiness as the policy anchor point. A good ecological environment and abundant material conditions are both sources of happiness for residents, and the blind pursuit of good ecology or economic growth will harm the welfare of residents. Therefore, while pursuing the “gold mountain”, the government should also pay attention to the protection of the “green mountain”. Secondly, when assessing environmental indicators, the government should focus on the combination of pollution stock and increment so as to effectively reduce the stock and curb the increment. This can strengthen the treatment of moderate to heavy air pollution, vigorously promote industrial upgrading and economic transformation, encourage innovative green technologies, develop an ecological economy and a green economy, accelerate the construction of ecological civilization, and build a beautiful China. Thirdly, the government needs to take objective environmental management performance as the basis, pay attention to the differences in residents’ perception and sensitivity to air quality, and take different measures for different groups. The elderly and low-income groups lack sensitivity to air pollution, have insufficient concern for the environment, and have weak pollution awareness, which is not conducive to the government’s promotion of environmental protection policies. For those who are not sensitive to pollution, the government should focus on improving residents’ knowledge of environmental protection, building their awareness of environmental protection, and cultivating their responsibility for environmental protection, so that they can effectively drive their environmental protection behavior and work together to build a green home; the young and high-income groups are prone to be overly sensitive to environmental pollution, generating excessive negative emotions or even overly aggressive actions, underestimating the government’s environmental management performance, and reducing their trust in the government. For the over-sensitive groups, the government should make environmental information open and transparent, strengthen the publicity of environmental performance, make pollution control a “visible control”, and build people’s confidence in the government’s environmental control.

### 6.3. Limitations and Future Work

First, although the data in 2016 is the latest data disclosed by CLDS to the public, it has been a certain number of years and lacks timeliness. Therefore, after the updated data is disclosed, the updated data can be used in the future to further verify and improve the conclusions of this paper. Second, this paper uses the PM10 concentration to represent the air pollution level, but air quality is affected by the concentration of multiple pollutants, and using a single pollutant to respond to air quality is biased. Again, this paper constructs pollution sensitivity indicators by using the difference between subjective and objective pollution levels, and future research can construct pollution sensitivity indicators by calculating elasticity indices and other ways to verify the moderating effect of pollution sensitivity on the relationship between air pollution and well-being. Finally, this paper only considers the moderating effect of pollution sensitivity, and future research can examine the moderating effect of other factors on air pollution and well-being to understand the relationship between air pollution and well-being in a more complete perspective.

## Figures and Tables

**Table 1:** Descriptive statistics.

Variable	Variable Description	Mean	Standard	Min	Max
Individual Level Variables					
Happiness	Ordinal variable 1–5	3.858	0.914	1.000	5.000
Age	Continuous variable	45.374	14.281	11.000	96.000
Age-squared/100	Continuous variable	22.628	12.516	1.212	92.163
Gender	Male = 0, female = 1	0.537	0.497	0.000	1.000
Education level	Ordinal variable 1–8	3.273	1.403	1.000	8.000
Marital status	Married = 1, other = 0	0.808	0.386	0.000	1.000
Religious belief	Yes = 1, No = 0	0.121	0.333	0.000	1.000
Pension	Yes = 1, No = 0	0.647	0.484	0.000	1.000
Medical insurance	Yes = 1, No = 0	0.892	0.311	0.000	1.000
Personal income (Log)	Continuous variable (Yuan)	10.000	1.247	4.606	14.952
Household registration	Rural = 1, urban = 0	0.702	0.462	0.000	1.000
Social trust	Ordinal variable 1–5	3.658	0.856	1.000	5.000
Healthy	Ordinal variable 1–5	3.697	1.000	1.000	5.000
City Level Variables					
The population density (Log)	Total population/area (Person/square kilometer)	6.353	0.637	2.892	7.824
GDP per capital (Log)	Continuous variable (Yuan/person)	11.153	0.463	10.081	11.968
Public expenditure ratio	Fiscal expenditure/GDP (%)	0.164	0.051	0.089	1.702
PM10	Continuous variable (µg/m^3^)	90.268	28.962	39.000	164.000
Pollution stock sensitivity	Continuous variable	−0.094	0.368	−0.904	0.981
Pollution incremental sensitivity	Continuous variable	−0.013	0.323	−0.897	0.872

**Table 2:** Effects of objective air pollution on residents’ happiness.

Variables	Happiness		
	Model 1	Model 2	Model 3
PM10	0.001 **	0.008 **	0.003 ***
	(0.001)	(0.003)	(0.001)
PM10^2^		−3.342 × 10^−5^ **	
		(1.623 × 10^−5^)	
PM10 (PM10 > PM10 *)			−0.007 ***
			(0.002)
Age	−0.066 ***	−0.066 ***	−0.066 ***
	(0.007)	(0.007)	(0.007)
Age squared/100	0.072 ***	0.072 ***	0.072 ***
	(0.008)	(0.008)	(0.008)
Gender	0.119 ***	0.118 ***	0.119 ***
	(0.027)	(0.027)	(0.027)
Married	0.310 ***	0.311 ***	0.310 ***
	(0.043)	(0.043)	(0.043)
Household registration	−0.049	−0.048	−0.046
	(0.034)	(0.034)	(0.034)
Religious belief	0.0423	0.049	0.049
	(0.0390)	(0.039)	(0.039)
Personal income (Log)	0.042 ***	0.042 ***	0.043 ***
	(0.015)	(0.015)	(0.015)
Pension	0.032	0.034	0.032
	(0.031)	(0.031)	(0.031)
Medical insurance	0.097 **	0.100 **	0.101 **
	(0.043)	(0.043)	(0.043)
Social trust	0.170 ***	0.169 ***	0.168 ***
	(0.016)	(0.016)	(0.016)
Education level	0.069 ***	0.068 ***	0.069 ***
	(0.014)	(0.014)	(0.014)
Healthy	0.256 ***	0.255 ***	0.254 ***
	(0.014)	(0.014)	(0.014)
GDP per capital (Log)	0.042	0.037	0.032
	(0.039)	(0.039)	(0.039)
Public expenditure ratio	0.351	0.376	0.325
	(0.274)	(0.274)	(0.274)
Public expenditure ratio	−0.044 *	−0.030	−0.029
	(0.024)	(0.025)	(0.025)
Observations	7143	7143	7143
Pseudo R^2^	0.042	0.043	0.043

Note: ***, ** and * indicate significance at 1%, 5% and 10%, respectively. Robust standard errors at the firm level are reported in parentheses. Same in the following table.

**Table 3:** Regression results of the moderating effect.

Variables	Happiness			
	Model 1	Model 2	Model 3	Model 4
PM10	0.009 ***	0.009 ***	−0.006 *	−0.005 *
	(0.003)	(0.003)	(0.003)	(0.003)
PM10^2^	−3.622 × 10^−5^ **	−3.373 × 10^−5^ **		
	(1.701 × 10^−5^)	(1.362 × 10^−5^)		
Stock sensitivity	−0.049		0.675 **	
	(0.040)		(0.296)	
Stock sensitivity × PM10	0.023 ***		−0.020 ***	
	(0.007)		(0.007)	
Stock sensitivity × PM10^2^	−1.223 × 10^−4^ ***			
	(3.642 × 10^−5^)			
Incremental sensitivity		−0.014		0.233
		(0.037)		(0.288)
Incremental sensitivity × PM10		0.037 ***		−0.012 *
		(0.008)		(0.007)
Incremental sensitivity × PM10^2^		−1.952 × 10^−4^ ***		
		(4.304 × 10^−5^)		
Individual characteristic variables	Control	Control	Control	Control
Urban characteristic variables	Control	Control	Control	Control
Observations	7143	7143	1912	1912
Pseudo R^2^	0.014	0.014	0.049	0.023

Note: ***, ** and * indicate significance at 1%, 5% and 10%, respectively.

**Table 4:** Age heterogeneity analysis.

Variables	Low Age				High Age	
	Model 1	Model 2	Model 3	Model 4	Model 5	Model 6
PM10	0.011 **	0.014 ***	0.012 ***	0.013 ***	−0.001	0.003
	(0.004)	(0.004)	(0.004)	(0.005)	(0.005)	(0.004)
PM10^2^	−4.774 × 10^−5^ **	−6.323 × 10^−5^ ***	−4.437 × 10^−5^ **	−5.562 **	5.233 × 10^−6^	1.456 × 10^−6^
	(2.992 × 10^−5^)	(2.282 × 10^−5^)	(2.116 × 10^−5^)	(2.563 × 10^−5^)	(2.601 × 10^−5^)	(2.181 × 10^−5^)
Stock sensitivity		0.129 **			−0.226 ***	
		(0.057)			(0.058)	
Stock sensitivity × PM10		0.028 ***			0.020 *	
		(0.010)			(0.011)	
Stock sensitivity × PM10^2^		−1.463 × 10^−4^ ***			−9.733 × 10^−5^ *	
		(5.142 × 10^−5^)			(2.602 × 10^−5^)	
Incremental sensitivity			0.086			−0.259 ***
			(0.056)			(0.057)
Incremental sensitivity × PM10			0.045 ***			0.059 ***
			(0.012)			(0.013)
Incremental sensitivity × PM10^2^			−2.402 × 10^−4^ ***			−2.942 × 10^−4^ ***
			(6.353 × 10^−5^)			(6.803 × 10^−5^)
Individual characteristic variables	Control	Control	Control	Control	Control	Control
Urban characteristic variables	Control	Control	Control	Control	Control	Control
Observations	3421	3421	3421	3722	3722	3722
Pseudo R^2^	0.020	0.020	0.023	0.017	0.010	0.015

Note: ***, ** and * indicate significance at 1%, 5% and 10%, respectively.

**Table 5:** Gender Heterogeneity Analysis.

Variables	Male				Female	
	Model 1	Model 2	Model 3	Model 4	Model 5	Model 6
PM10	0.016 ***	0.0145 ***	0.019 ***	0.010 **	0.003	0.009 *
	(0.005)	(0.005)	(0.004)	(0.004)	(0.005)	(0.005)
PM10^2^	−7.482 × 10^−5^ ***	−6.223 × 10^−5^ **	−8.932 × 10^−5^ ***	−4.152 × 10^−5^ *	−1.312 × 10^−5^	−4.804 × 10^−5^ *
	(2.501 × 10^−5^)	(2.572 × 10^−5^)	(2.403 × 10^−5^)	(2.311 × 10^−5^)	(2.293 × 10^−5^)	(2.761 × 10^−5^)
Stock sensitivity		−0.052			−0.047	
		(0.059)			(0.055)	
Stock sensitivity × PM10		0.005			0.039 ***	
		(0.010)			(0.010)	
Stock sensitivity × PM10^2^		−2.437 × 10^−5^			−2.023 × 10^−4^ ***	
		(5.368 × 10^−5^)			(4.972 × 10^−5^)	
Incremental sensitivity			0.014			0.076
			(0.062)			(0.060)
Incremental sensitivity × PM10			−0.007			0.061 ***
			(0.014)			(0.014)
Incremental sensitivity × PM10^2^			2.923 × 10^−5^			−2.993 × 10^−4^ ***
			(7.154 × 10^−5^)			(7.142 × 10^−5^)
Individual characteristic variables	Control	Control	Control	Control	Control	Control
Urban characteristic variables	Control	Control	Control	Control	Control	Control
Observations	3365	3365	3365	3778	3778	3778
Pseudo R^2^	0.016	0.015	0.010	0.016	0.014	0.041

Note: ***, ** and * indicate significance at 1%, 5% and 10%, respectively.

**Table 6:** Income Heterogeneity Analysis.

Variables	Low Income				High Income	
	Model 1	Model 2	Model 3	Model 4	Model 5	Model 6
PM10	0.012 *	−0.005	0.004	0.016 ***	0.011 **	0.013 ***
	(0.006)	(0.006)	(0.006)	(0.005)	(0.005)	(0.004)
PM10^2^	−5.302 × 10^−5^ *	3.301 × 10^−5^	−1.023 × 10^−5^	−7.183 × 10^−5^ ***	−5.637 × 10^−5^ **	−5.588 × 10^−5^ **
	(3.213 × 10^−5^)	(3.113 × 10^−5^)	(2.872 × 10^−5^)	(2.771 × 10^−5^)	(2.417 × 10^−5^)	(2.349 × 10^−5^)
Stock sensitivity		−0.130 **			−0.269 ***	
		(0.069)			(0.058)	
Stock sensitivity × PM10		0.027 **			0.030 ***	
		(0.012)			(0.010)	
Stock sensitivity × PM10^2^		−1.234 × 10^−4^ **			−1.683 × 10^−4^ ***	
		(6.132 × 10^−5^)			(5.384 × 10^−5^)	
Incremental sensitivity			−0.036			−0.165 ***
			(0.067)			(0.056)
Incremental sensitivity × PM10			0.050 ***			0.028 **
			(0.016)			(0.013)
Incremental sensitivity × PM10^2^			−2.418 × 10^−2^ ***			−1.478 × 10^−4^ **
			(8.266 × 10^−5^)			(6.658 × 10^−5^)
Individual characteristic variables	Control	Control	Control	Control	Control	Control
Urban characteristic variables	Control	Control	Control	Control	Control	Control
Observations	3058	3058	3058	4085	4085	4085
Pseudo R^2^	0.021	0.021	0.021	0.020	0.023	0.022

Note: ***, ** and * indicate significance at 1%, 5% and 10%, respectively.

**Table 7:** Variable substitution test.

Variables	Happiness		Satisfaction		
	Model 1	Model 2	Model 3	Model 4	Model 5
PM10			0.009 ***		
			(0.003)		
PM10^2^			−4.393 × 10^−5^ ***		
			(1.702 × 10^−5^)		
SO_2_	0.015 ***			0.017***	
	(0.003)			(0.003)	
SO_2_^2^	−1.113 × 10^−4^ ***			−1.713 × 10^−4^ ***	
	(3.886 × 10^−4^)			(3.812 × 10^−5^)	
NO_2_		0.028 ***			0.034 ***
		(0.011)			(0.011)
NO_2_^2^		−3.412 × 10^−4^ ***			−4.087 × 10^−4^ ***
		(1.304 × 10^−4^)			(1.302 × 10^−4^)
Individual characteristic variables	Control	Control	Control	Control	Control
Urban characteristic variables	Control	Control	Control	Control	Control
Pseudo R^2^	0.020	0.025	0.041	0.015	0.026
Observations	6607	7700	7143	6607	7700

Note: *** indicate significance at 1%.

**Table 8:** Model substitution test and bilateral abbreviated tail test.

Variables	Model 1	Model 2	Model 3
	OLogit	Regress	OProbit
PM10	0.014 **	0.007 ***	0.009 **
	(0.006)	(0.003)	(0.005)
PM10^2^	−6.023 × 10^−5^ **	−3.022 × 10^−5^ **	−3.404 × 10^−5^ *
	(2.852 × 10^−5^)	(1.323 × 10^−5^)	(2.062 × 10^−5^)
Observations	7143	7143	5911
Pseudo R^2^	0.043	0.105	0.007

Note: ***, ** and * indicate significance at 1%, 5% and 10%, respectively.

## Data Availability

Not applicable.
